# Discrepancies in isoniazid susceptibility profiles: Bactec MGIT 960-resistant but GenoType MTBDR*plus*-susceptible *Mycobacterium tuberculosis* strains in Hunan, China

**DOI:** 10.1128/spectrum.01101-25

**Published:** 2025-10-01

**Authors:** Zhenhua Chen, Peilei Hu, Jingwei Guo, Jue Wang, Binbin Liu, Yunhong Tan

**Affiliations:** 1 Clinical Laboratory, Hunan Chest Hospital, Changsha, People's Republic of China; End TB Dx Consulting LLC, San Diego, California, USA

**Keywords:** *Mycobacterium tuberculosis*, drug susceptibility testing, Bactec MGIT 960, Genotype MTBDR*plus*, isoniazid, whole-genome sequencing

## Abstract

Discordant drug susceptibility testing (DST) results between the Bactec MGIT 960 system (MGIT) and the GenoType MTBDR*plus* assay (MTBDR*plus*) for isoniazid (INH) complicate clinical decision-making. In this study, we performed minimum inhibitory concentration (MIC) assays and whole-genome sequencing (WGS) on 53 *Mycobacterium tuberculosis* strains identified as INH-resistant by MGIT but INH-susceptible by MTBDR*plus*. The variants conferring INH resistance were evaluated by the WHO mutation catalogue. Our results showed that only five strains carried variants classified as “associated with resistance” (Group 1/2), including *katG* Trp39STOP, *katG* Ser315Asn, *inhA* −154G>A, and *inhA* Ser94Ala. In addition, 44 strains carried 70 variants classified as “Group 3: Uncertain significance” across nine genes, including *katG*, *ahpC*, *inhA*, *Rv0010c*, *Rv1129c*, *Rv2752c*, *mshA*, *dnaA*, and *Rv1258c*. The remaining four strains carried no variants (Groups 1–3) linked to INH resistance. No significant difference in the prevalence of high-level INH resistance was observed between lineage 2 and lineage 4 strains (*χ*² = 0.232, *P* = 0.630). Our findings indicate that the variants classified as “uncertain significance” may be the main genetic determinants causing discordant results, highlighting their associations with INH resistance that need to be further investigated.

**IMPORTANCE:**

This study addresses a critical challenge in drug susceptibility testing (DST): the discrepancies in DST results for isoniazid (INH) between the Bactec MGIT 960 system and the GenoType MTBDR*plus* assay. These discordant results significantly complicate treatment decisions, potentially leading to suboptimal patient outcomes. Using MIC assays and WGS on 53 clinical *Mycobacterium tuberculosis* strains, we provide valuable insights into the genetic basis of INH resistance. Our findings showed that only a small fraction of strains carried variants definitively linked to INH resistance, while a larger number harbored variants of uncertain significance across multiple genes, underscoring the complexity of INH resistance mechanisms. This study highlights the urgent need to refine our understanding of these “Group 3: uncertain significance” variants, as they appear to be a primary driver of the discrepancies. Additionally, this study emphasizes the importance of integrating advanced sequencing tools into DST to improve the accuracy of INH resistance detection.

## INTRODUCTION

Isoniazid (INH), a cornerstone of first-line tuberculosis (TB) treatment due to its potent early bactericidal activity, has increasingly shown resistance worldwide. INH resistance, whether occurring alone or in combination with other drugs, has become the most prevalent form of anti-TB drug resistance (1–3). A global surveillance data set reveals significant variations in the prevalence of INH resistance across different regions, ranging from 14% in West/Central Europe and Africa to 30% in Eastern Europe during the period of 1994–2009 (1). Between 2003 and 2017, the global prevalence of INH-resistant, rifampin-susceptible TB (Hr-TB) was 7.4% among new TB cases and 11.4% among previously treated cases, based on data from 156 countries or territories involving 211,753 patients (2). A retrospective cohort study conducted in eastern China (2013–2018) further underscored the clinical significance of Hr-TB, reporting a prevalence of 4.6% (63/1,359) among TB patients (3). Given the poorer treatment outcomes associated with INH-resistant TB compared to drug-susceptible TB (4, 5), the rapid and accurate detection of INH resistance is critical for effective TB management.

The Bactec MGIT 960 system (MGIT) and Genotype MTBDR*plus* assay (MTBDR*plus*) are widely used, WHO-recommended methods for DST in the tuberculosis laboratory. The MGIT, a phenotypic DST (pDST) method, serves as the reference standard for detecting INH resistance but is time-consuming and labor-intensive. In contrast, the MTBDR*plus*, a genotypic DST (gDST) method, offers rapid detection of INH resistance by identifying variants in the *katG* gene and the promoter region of *inhA* genes, achieving high specificity (>95%) but variable sensitivity (75%–94%) (6, 7). These differences can lead to discrepancies between the two tests, challenging clinical decision-making. For instance, MTBDR*plus* may indicate susceptibility to INH, while MGIT detects resistance, creating potential conflicts in interpretation (8, 9).

Several factors may contribute to these discrepancies between MTBDR*plus* and MGIT. First, while MTBDR*plus* detects INH resistance-associated mutations in the *katG* gene and *inhA* promoter region, certain resistance-conferring variants within these regions are not covered by the MTBDR*plus* probes, potentially leading to false-susceptible results (10, 11). Furthermore, in cases of INH heteroresistance, where patients harbor mixed populations of INH-susceptible and INH-resistant *Mycobacterium tuberculosi*s (MTB), conventional DST remains the most sensitive method for detection, whereas the MTBDR*plus* lacks the analytical sensitivity to reliably detect resistant subpopulations at the clinically relevant threshold of 1% (12).

Although previous studies have investigated the inconsistencies between pDST and gDST in detecting INH resistance (8, 11, 12), the genetic determinants responsible for strains reported as INH-resistant by MGIT but INH-susceptible by MTBDR*plus* remain incompletely understood. This study, therefore, aims to investigate the genetic determinants driving these discrepancies by integrating clinical data, MIC testing, and WGS of these specific clinical MTB isolates, with the potential to uncover novel genetic determinants contributing to these discordant results.

## MATERIALS AND METHODS

### Study design

Between October 2014 and September 2021, we enrolled MTB-positive cases identified by MGIT culture from TB patients previously classified as INH-resistant by MGIT but INH-susceptible by the MTBDR*plus*. Relevant sociodemographic and clinical data, including gender, age, treatment history, infection sites, and biological samples, were collected. If multiple MTB strains were isolated from the same patient, only the first strain isolated during the study period was included. All strains were stored at −80°C in tryptic soy broth with 10% glycerol before use. To ensure accuracy, strains were retested using both MGIT and MTBDR*plus* for DST confirmation, followed by WGS and MIC, to investigate the basis of the discordant results.

### DST by MGIT

Bacterial growth from Middlebrook 7H10 medium was used to prepare the inoculum. After incubation at 36 ± 1°C for approximately 21 days, fresh colonies were collected using a sterile 10 µL loop. These colonies were then transferred into a sterile bottle containing glass beads and 100 µL of 0.05% Tween 80. The bacterial clumps were dispersed by vortexing for 30 seconds and suspended in 3 mL of saline. After settling for 10–15 minutes, the upper 0.5 mL was used to measure bacterial concentration, adjusted to a McFarland turbidity of 0.5, and diluted 1:5 in sterile saline. The prepared inoculum was used for MGIT INH DST according to the manufacturer’s instructions for the Bactec MGIT 960 SIRE Kit (Becton, Dickinson and Company, Sparks, MD, USA), with a critical isoniazid (INH) concentration of 0.1 µg/mL. Quality control was conducted using MTB H37Rv (ATCC 27294) in each test batch.

### DST by MTBDR*plus*

The MTBDR*plus* assay was performed on the recovered MTB strains according to the manufacturer’s instructions (Hain Lifescience, Germany). The procedure involved a series of steps, including DNA extraction, preparation of the master mix, multiplex PCR amplification, and reverse hybridization. Throughout the experimental process, sterile nuclease-free water was used as the negative control, and all steps were conducted in separate rooms to prevent cross-contamination. A result was considered valid if all expected control bands were present; otherwise, it was labeled as invalid. The absence of at least one wild-type band or the presence of bands indicating a variant in each drug resistance–related gene suggested that the strain was resistant to a specific antibiotic. In contrast, if all wild-type probes of a gene stained positive and no detectable variants were found within the examined region, the strain was deemed susceptible to the respective antibiotic.

### MIC

The MIC-based pDST for INH was conducted using Middlebrook 7H9 Broth Microdilution (BMD) plates, following WHO guidelines (13). Each strain was tested in duplicate on BMD plates, with MTB H37Rv serving as the control. Briefly, the recovered strains were harvested from Middlebrook 7H10 medium, suspended in saline–Tween with glass beads for agitation, and the supernatant turbidity was standardized to 0.5 McFarland. The bacterial suspension was then diluted 1:100 with Middlebrook 7H9 broth supplemented with 10% oleic acid–albumin–dextrose–catalase (OADC). Subsequently, 100 µL of this mixture was added to the wells of a 96-well cell culture plate containing dried INH (BASO Company, Zhuhai, China). INH concentrations in Middlebrook 7H9 medium were as follows: 0.03, 0.06, 0.125, 0.25, 0.5, 1.0, 2.0, 4.0, 8.0, and 16.0 µg/mL. Quality control for each strain included one negative growth control well and two positive control wells (100% and 1% inoculum of the 0.5 McFarland standard suspension) in drug-free control wells. Plates were sealed with plastic covers and incubated at 37°C in ambient air for 7 to 21 days. Readings were performed once the 1% and 100% control wells exhibited visible growth, while the negative control did not. The MIC was determined as the lowest concentration at which no visual growth was observed. Strains with a MIC greater than or equal to 1  µg/mL were classified as exhibiting high-level resistance to INH (14), whereas those with MICs ranging from 0.125 to 0.5  µg/mL (inclusive) were classified as having low-level resistance to INH.

### WGS and bioinformatics analysis

Extraction and purification of genomic DNA were carried out following the Bacterial DNA Extraction Kit (Gene-Optimal, 60,300K-50 T) protocols. All WGS procedures were performed by Shanghai Gene-Optimal Science & Technology (Shanghai, China). DNA libraries were prepared using the FS DNA Library Prep Kit V6 (RK20259) on the Illumina platform. Indexed libraries were pooled and loaded onto an Illumina HiSeq instrument according to the manufacturer’s instructions (Illumina, San Diego, CA, USA). Paired-end 150 bp (PE150) sequencing was performed on the Illumina NovaSeq 6000 platform following library quality control. Raw FASTQ sequences were filtered using Fastp (v0.20.0) to remove adapter sequences, duplicate reads, and low-quality reads. The cleaned reads were aligned to the MTB H37Rv reference genome (NC_000962.3) using BWA (v0.7.17) and SAMtools (v1.7). Single-nucleotide polymorphisms (SNPs) were called using Freebayes (v1.3.2) with a minimum coverage of 10 reads and a mapping quality score ≥100. An in-house script was used to extract SNP sequences and calculate pairwise distances between samples. The core genome was obtained using the snippy-core plugin (v4.4.3) after masking short tandem repeat genes (e.g., PE/PPE). Multiple sequence alignment was performed with Gubbins (v2.4.1), followed by reconstruction of the maximum likelihood (ML) phylogenetic tree using IQ-TREE (v2.1.4). INH resistance-associated variants were primarily identified by comparison with the WHO mutation catalogue (15). Additional resistance-associated variants not yet included in the WHO catalogue, as well as strain lineages, were identified by analyzing BAM files using TB-Profiler (v6.3.0, https://github.com/jodyphelan/TBProfiler).

### Statistical analysis

All statistical analyses were performed using IBM SPSS (version 20.0; IBM Corporation, Armonk, NY, USA). The Pearson chi-squared test was used to assess associations between strain lineages and resistance levels. In addition, an independent two-tailed Student’s *t*-test was performed to assess the relationship between the number of genetic variants and resistance levels. A *P*-value less than 0.05 was considered statistically significant.

## RESULTS

### Patient demographics

**Fig 1:**
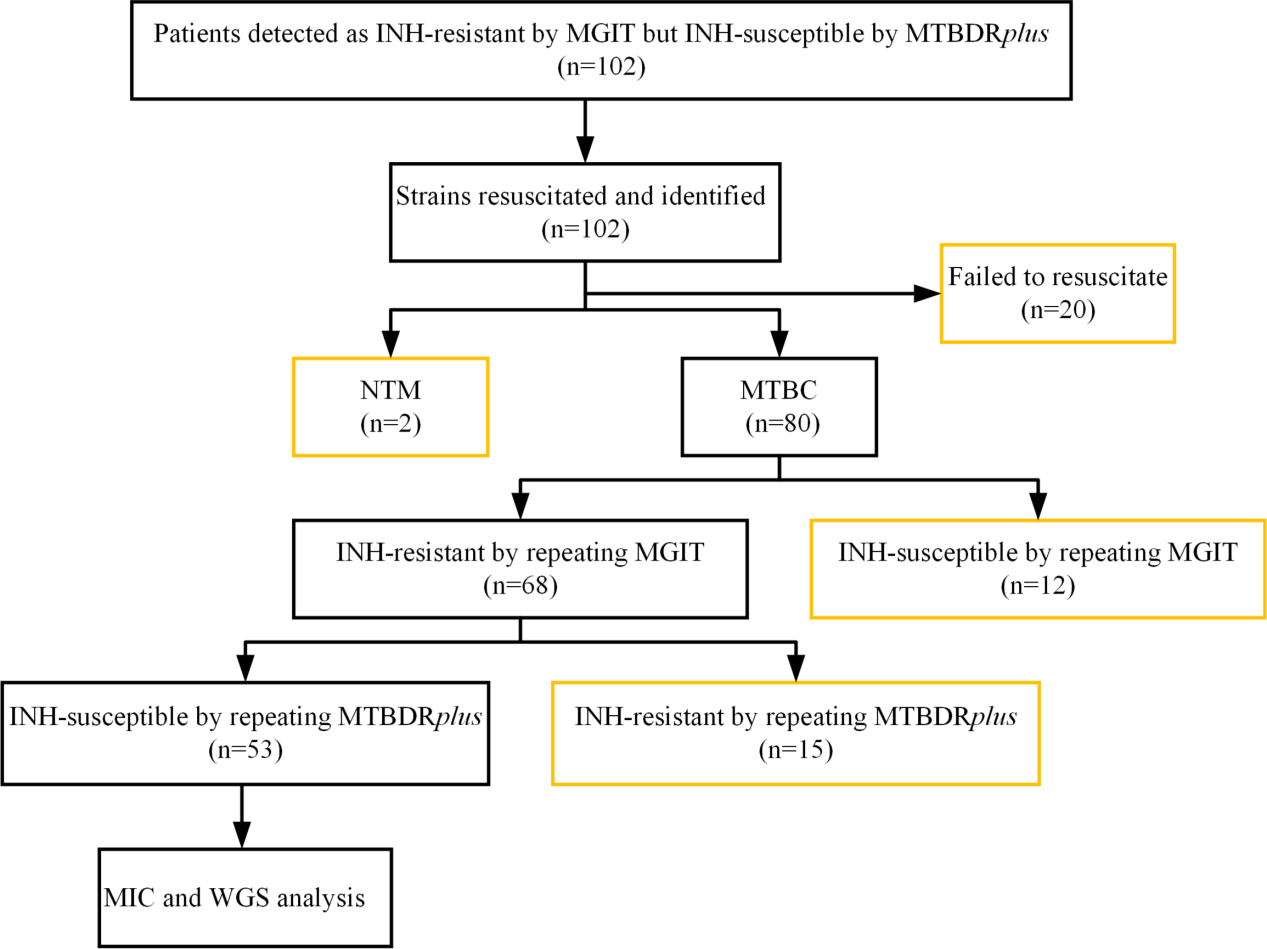
Study flowchart. Orange outlines of boxes indicate excluded cases. INH, isoniazid. NTM, nontuberculous mycobacteria. MTBC, *Mycobacterium tuberculosis* complex. MIC, minimum inhibitory concentration. WGS, whole-genome sequencing.

Of the 102 patients classified as INH-resistant by MGIT but INH-susceptible by MTBDR*plus*, a total of 53 patients were included for analysis, including 41 males (77.4%, 41/53) and 12 females (22.6%, 12/53). The median age was 52 years (interquartile range: 46–62 years). Among the patients, five (9.4%, 5/53) were new cases, and 48 (90.6%, 48/53) were retreated cases. Multidrug-resistant tuberculosis (MDR-TB) was identified in 40 cases (75.5%, 40/53). Of the strains, 49 (92.5%, 49/53) were isolated from sputum samples, while the remaining 4 (7.5%, 4/53) were obtained from bronchoalveolar lavage fluid. A total of 49 patients were excluded from the study due to a variety of reasons (Fig. 1)

### Lineage and genetic distance based on WGS

**Fig 2:**
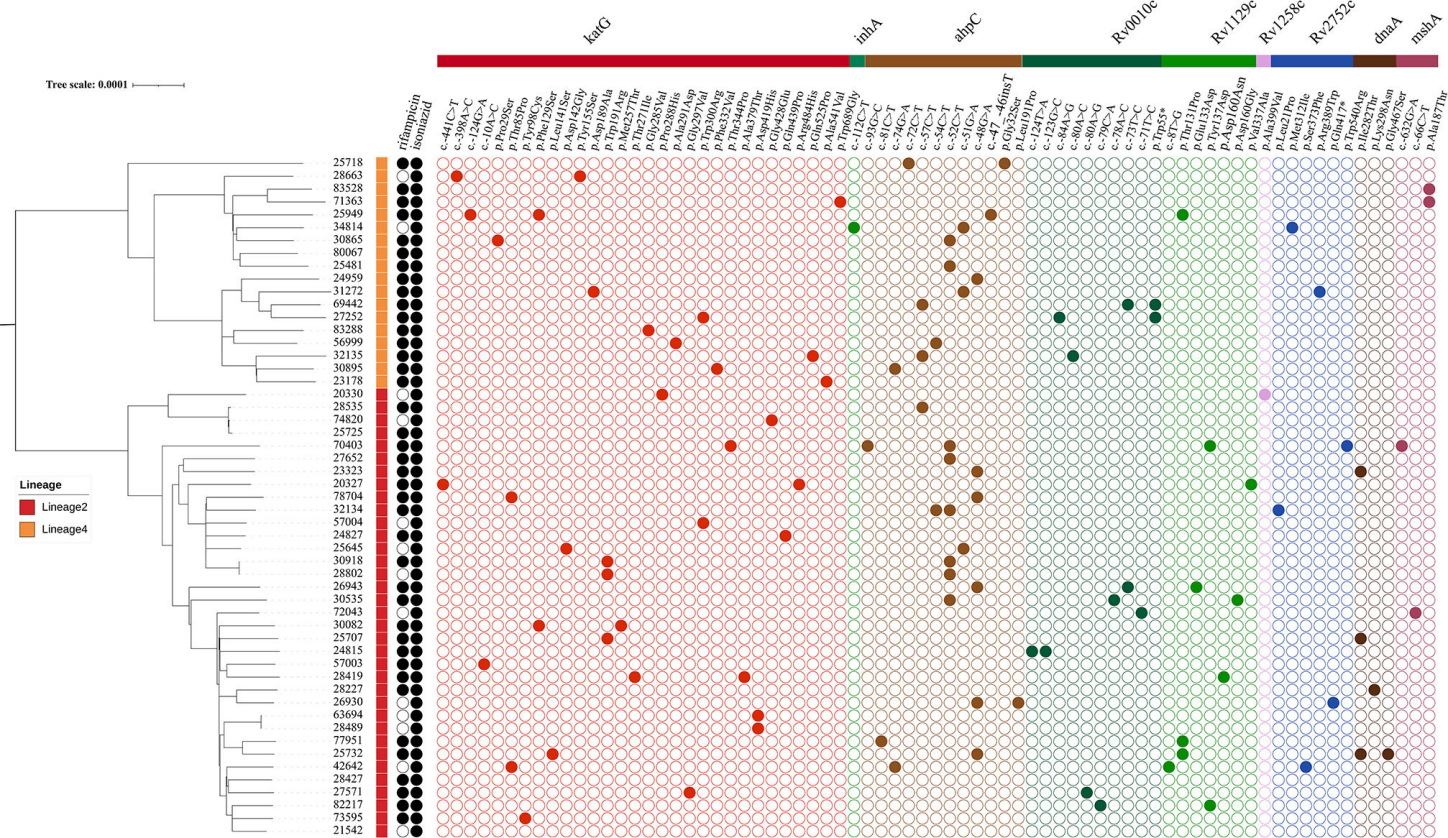
Phylogenetic analysis of 53 *Mycobacterium tuberculosis* strains with corresponding drug resistance profiles and mutations in Group 3: uncertain significance. Black solid circles denote resistant phenotypes tested by MGIT 960, while black open circles indicate susceptible phenotypes. Colored solid circles indicate the presence of a mutation.

Phylogenetic analysis based on WGS for 53 MTB strains revealed two major lineages, lineage 2 and lineage 4 (Fig. 2). A total of 35 strains (66.0%, 35/53) belonged to the predominant lineage 2 (mainly East Asian), including 31 (58.5%, 31/53) sublineage 2.2.1 strains and 4 (7.5%, 4/53) sublineage 2.2.2 strains. Eighteen (34.0%, 18/53) strains belonged to lineage 4 (mainly Euro-American), including 1 (1.9%, 1/53) sublineage 4.2.2 strain, 7 (13.2%, 7/53) sublineage 4.4.2 strains, 1 (1.9%, 1/53) sublineage 4.4 strain, and 9 (17.0%, 9/53) sublineage 4.5 strains.

Pairwise genetic distance analysis classified six strains into three clusters (clustering rate: 11.3%, 6/53), based on a genetic distance threshold of fewer than 12 SNPs. All clustered strains belonged to lineage 2. Among these clustered strains, two were isolated from new TB patients, while four were from previously treated patients.

### MIC distribution

**Fig 3:**
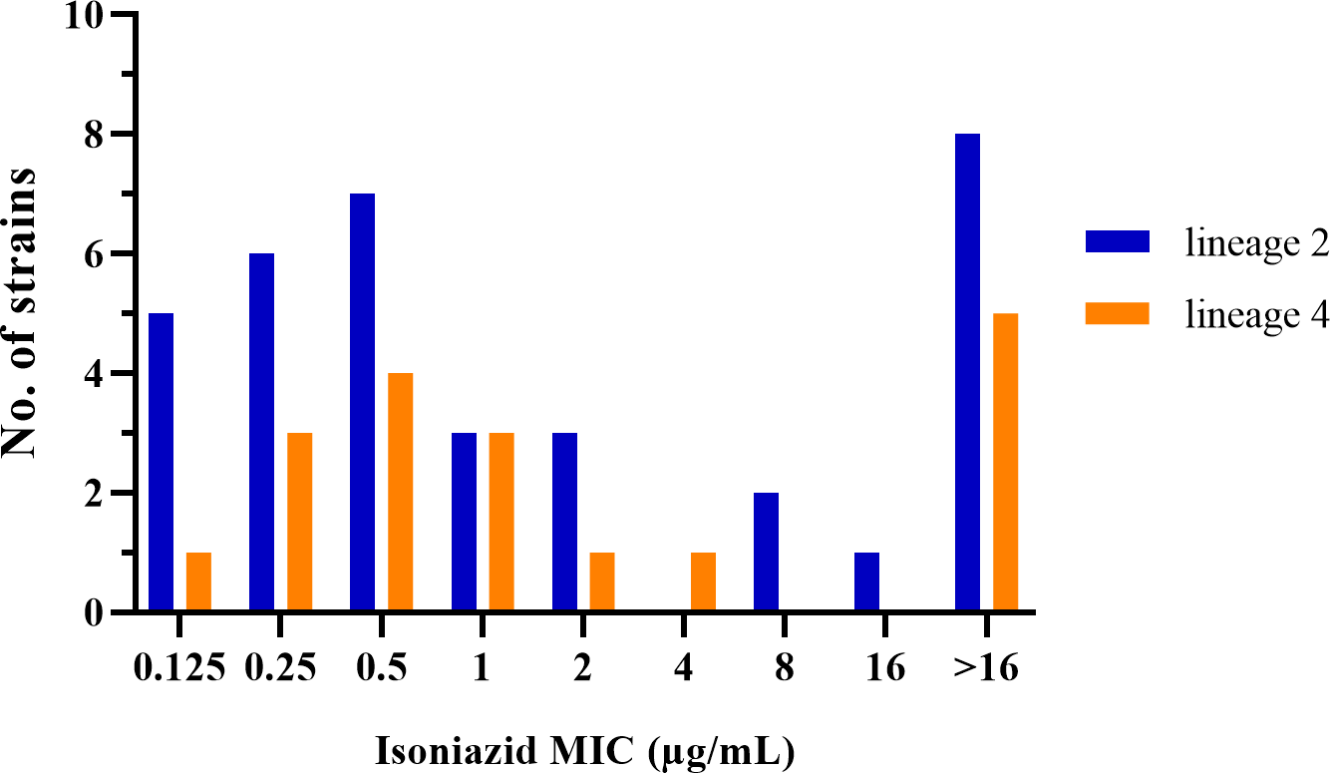
Minimum inhibitory concentration distributions of isoniazid by lineage for 53 *Mycobacterium tuberculosis* strains exhibiting discordant susceptibility profiles (INH-resistant by MGIT 960 but INH-susceptible by MTBDR*plus*).

The median MIC for INH among the 53 strains was 1 µg/mL. High-level INH resistance (MIC ≥1 µg/mL) was observed in 27 strains (50.9%, 27/53), with 13 strains exceeding 16 µg/mL. Specifically, lineage 2 strains exhibited a median MIC of 0.5 µg/mL, with 17 (48.6%, 17/35) showing high-level resistance, while lineage 4 strains had a median MIC of 1 µg/mL, with 10 (55.6%, 10/18) showing high-level resistance. No significant difference in the prevalence of high-level INH resistance was observed between two lineages (*χ*² = 0.232, *P* = 0.630) (Fig. 3).

### Variants associated with INH resistance in 53 strains

In total, 83 variants were identified across nine genes associated with INH resistance, including *katG*, *ahpC*, *inhA*, *Rv0010c*, *Rv1129c*, *Rv2752c*, *mshA*, *dnaA*, and *Rv1258c*. Of the 83 variants, all but one variant (*katG* Val1Ala) were included in the WHO catalogue. The 82 catalogue-listed variants were categorized as follows: two (*katG* Ser315Asn and *inhA* −154G>A) in group 1, two (*katG* Trp39STOP and *inhA* Ser94Ala) in group 2, 73 in group 3, one in group 4, and four in group 5.

Among the 53 strains, only five carried variants classified as “associated with resistance” (Groups 1 and 2), including *katG* Trp39STOP, *katG* Ser315Asn, *inhA* −154G>A, and *inhA* Ser94Ala (Table 1). The frequency of *katG* Trp39STOP and *katG* Ser315Asn was 98.8% and 42.0%, respectively. In addition, 44 strains carried 70 variants classified as “uncertain significance” (Group 3) (Table 2). The remaining four strains lacked variants from Groups 1 to 3 but carried only variants classified as “not associated with resistance” (Groups 4 and 5) (Table S1).

**TABLE 1:** Mutation profiles and INH MIC values of 5 INH-resistant MTB strains with variants in Groups 1 and 2^*a*^^,*g*^

Resistant level	Sample ID	*katG*	*inhA*	*ahpC*	*dnaA*	*mshA*	*Rv0010c*	*Rv1129c*	*Rv1258c*	MIC (µg/mL)
Low	25707	Trp191Arg^*d*^^,*f*^/Arg463Leu	−154G>A^*b*^^,*f*^	–^*h*^	Ile282Thr^*d*^^,*f*^	Ala187Val	–	−28T>C	Glu194fs	0.125
Low	28489	Asp419His^*d*^^,*f*^/Arg463Leu	Ser94Ala^*c*^^,*f*^	–	–	Ala187Val	–	−28T>C	Glu194fs	0.25
Low	63694	Arg463Leu/Asp419His^*d*^^,*f*^	Ser94Ala^*c*^^,*f*^	–	–	Ala187Val	–	−28T>C	Glu194fs	0.25
High	24959	Trp39STOP^*c*^^,*f*^	–	−48G>A^*d*^^,*f*^	–	–	–	–	–	>16
High	72043	Ser315Asn^*b*^^,*e*^/Arg463Leu	–	–	–	−66C>T^*d*^^,*f*^/Ala187Val	−71T>C^*d*^^,*f*^	−28T>C	Glu194fs	2

^*a*^ Variants were classified according to the WHO mutation catalogue (2023) into five groups.

^*b*^ Group 1: associated with resistance.

^*c*^ Group 2: associated with resistance–interim.

^*d*^ Group 3: uncertain significance.

^*e*^ Variant allele frequencies were categorized as 25%–75%.

^*f*^ >90%.

^*g*^ Unlabeled mutations represent Group 4 (not associated with resistance–interim) or Group 5 (not associated with resistance) variants with allele frequencies >90%. MIC, minimum inhibitory concentration; fs, frameshift.

^*h*^ "–" indicates that the mutation was not detected.

**TABLE 2:** Variant profiles and INH MIC values of 44 INH-resistant MTB strains without Group 1 and 2 variants but with Group 3 variants^*a*^^,^^*h*^

Resistant level	Sample ID	*katG*	*inhA*	*ahpC*	*dnaA*	*mshA*	*Rv0010c*	*Rv1129c*	*Rv1258c*	*Rv2752c*	MIC(µg/mL)
Low	20327	−441C>T^*b*^^,*f*^/Arg484His^*b*^^,*f*^/Arg463Leu	–^*i*^	–	–	–	–	Val337Alac^*b*^^,*e*^/−28T>C	Glu194fs	–	0.5
Low	20330	Pro288His^*b*^^,*f*^/Arg463Leu	–	–	–	–	–	−28T>C	Ala399Val^*b*^^,*f*^	–	0.125
Low	24815	Arg463Leu	–	–	–	Ala187Val	−123G>C^*b*^^,*f*^/−124T>A^*b*^^,*f*^	−28T>C	Glu194fs	–	0.5
Low	25481	–	–	−52C>T^*b*^^,*f*^	–	–	–	–	–	Met31Ile	0.5
Low	25718	–	–	Gly32Ser^*b*^^,*f*^/−72C>T^*b*^^,*f*^	–	–	–	–	–	–	0.25
Low	25732	Leu141Ser^*b*^^,*f*^/Arg463Leu	–	−48G>A^*b*^^,*f*^	Gly467Ser^*b*^^,*f*^/Ile282Thr^*b*^^,*f*^	Ala187Val	–	Thr131Pro^*b*^^,*f*^/−28T>C	Glu194fs	–	0.25
Low	25949	−124G>A^*b*^^,*f*^/Phe129Ser^*b*^^,*f*^	–	−47_−46insT^*b*^^,*f*^	–	–	–	Thr131Pro^*b*^^,*f*^	–	Met31Ile	0.125
Low	27252	Trp300Arg^*b*^^,*f*^	–	–	–	–	Trp55STOP^*b*^^,*f*^/−84A>G^*b*^^,*f*^	–	–	–	0.5
Low	28663	−398A>C^*b*^^,*f*^/Tyr155Ser^*b*^^,*f*^	–	–	–	–	–	–	–	Met31Ile	0.25
Low	28802	Trp191Arg^*b*^^,*f*^/Arg463Leu	–	−52C>T^*b*^^,*f*^	–	–	–	−28T>C	Glu194fs	–	0.125
Low	30082	Phe129Ser^*b*^^,*d*^/Met257Thr^*b*^^,*d*^/Arg463Leu	–	–	–	Ala187Val	–	−28T>C	Glu194fs	–	0.25
Low	30895	Phe332Val^*b*^^,*f*^	–	−74G>A^*b*^^,*f*^	–	–	–	–	–	–	0.25
Low	30918	Trp191Arg^*b*^^,*f*^/Arg463Leu	–	−52C>T^*b*^^,*f*^	–	–	–	−28T> C	Glu194fs	–	0.125
Low	32134	Arg463Leu/Val1Ala^*f*^^,*g*^	–	−52C>T^*b*^^,*f*^/−54C>T^*b*^^,*f*^	–	–	–	−28T>C	Glu194fs	Leu21Pro^*b*^^,*f*^	0.25
Low	57003	−10A>C^*b*^^,*f*^/Arg463Leu	–	–	–	Ala187Val	–	−28T>C	Glu194fs	–	0.5
Low	57004	Trp300Arg^*b*^^,*f*^/Arg463Leu	–	–	–	–	–	−28T>C	Glu194fs	–	0.5
Low	71363	Trp689Gly^*b*^^,*f*^	–	–	–	Ala187Thr^*b*^^,*f*,^	–	–	–	Met31Ile	0.5
Low	73595	Tyr98Cys^*b*^^,*f*^/Arg463Leu	–	–	–	Ala187Val	–	−28T>C	Glu194fs	–	0.25
Low	74820	Gly428Glu^*b*^^,*c*^/Arg463Leu	–	–	–	–	–	−28T>C	–	–	0.5
Low	78704	Thr85Pro^*b*^^,*f*^/Arg463Leu	–	−48G>A^*b*^^,*f*^	–	–	–	−28T>C	Glu194fs	–	0.125
Low	83288	Gly285Val^*b*^^,*f*^	–	–	–	–	–	–	–	–	0.5
High	23178	Ala541Val^*b*^^,*f*^	–	–	–	–	–	-	-	–	1
High	23323	Arg463Leu	–	−48G>A^*b*^^,*f*^	Ile282Thr^*b*^^,*f*^	–	–	−28T>C	Glu194fs	–	>16
High	24827	Gln439Pro^*b*^^,*f*^/Arg463Leu	–	–	–	–	–	−28T>C	Glu194fs	–	2
High	25645	Asp142Gly^*b*^^,*f*^/Arg463Leu	–	−51G>A^*b*^^,*f*^	–	–	–	−28T>C	Glu194fs	–	8
High	26930	Arg463Leu	–	−48G>A^*b*^^,*f*^/Leu191Pro^*b*^^,*f*^	–	Ala187Val	–	−28T>C	Glu194fs	Gln417STOP^*b*^^,*f*^	>16
High	26943	Arg463Leu	–	−48G>A^*b*^^,*f*^	–	Ala187Val	−73T>C^*b*^^,*f*^	Glu133Asp^*b*^^,*f*^/−28T>C	Glu194fs	–	16
High	27571	Gly297Val^*b*^^,*f*^/Arg463Leu	–	–	–	Ala187Val	−80A>G^*b*^^,*d*^	−28T>C	Glu194fs	–	1
High	27652	Arg463Leu	–	−52C>T^*b*^^,*f*^	–	–	–	−28T>C	Glu194fs	–	>16
High	28227	Arg463Leu	–	–	Lys298Asn^*b*^^,*f*^	Ala187Val	–	−28T>C	Glu194fs	–	>16
High	28419	Ala379Thr^*b*^^,*f*^/Thr271Ile^*b*^^,*f*^/Arg463Leu	–	–	–	Ala187Val	–	Asp160Asn^*b*^^,*f*^/−28T>C	Glu194fs	–	1
High	28535	Arg463Leu	–	−57C>T^*b*^^,*f*^	–	–	-	−28T>C	–	–	>16
High	30535	Arg463Leu	–	−52C>T^*b*^^,*f*^	–	Ala187Val	−78A>C^*b*^^,*f*^	Asp160Gly^*b*^^,*f*^/−28T>C	Glu194fs	–	>16
High	30865	Pro29Ser^*c*,*d*^	–	−52C>T^*c*,*d*^	–	–	–	–	–	Met31Ile	1
High	31272	Asp189Ala^*b*^^,*f*^	–	−51G>A^*b*^^,*f*^	–	–	–	–	–	Arg389Trp^*b*^^,*f*^	>16
High	32135	Gln525Pro^*b*^^,*f*^	–	−57C>T^*b*^^,*f*^	–	–	−80A>C^*b*^^,*c*^	–	–	–	2
High	34814	–	−112C>T^*b*^^,*f*^	−51G>A^*b*^^,*f*^	–	–	–	–	–	Met312Ile^*b*^^,*f*^–/Met31Ile	1
High	42642	Thr85Pro^*b*^^,*f*^/Arg463Leu	–	−74G>A^*b*^^,*f*^	–	Ala187Val	–	−8T>G^*b*^^,*f*^/−28T > C	Glu194fs	Ser373Phe^*b*^^,*f*^	1
High	56999	Ala291Asp^*b*^^,*f*^	–	−54C>T^*b*^^,*f*^	–	–	–	–	–	–	>16
High	69442	Val1Ala^*g*^^,*f*^	–	−57C>T^*b*^^,*f*^	–	–	−73T>C^*b*^^,*f*^/Trp55STOP^*b*^^,*f*^	–	–	–	>16
High	70403	Thr344Pro^*b*^^,*f*^/Arg463Leu	–	−93G>C^*b*^^,*f*^/−52C>T^*b*^^,*f*^	–	−632G>A^*b*^^,*f*^	–	Tyr137Asp^*b*^^,*f*^/−28T>C	Glu194fs	Trp540Arg^*b*^^,*f*^	8
High	77951	Arg463Leu	–	−81C>T^*b*^^,*f*^	–	Ala187Val	–	Thr131Pro^*b*^^,*f*^/−28T>C	Glu194fs	–	>16
High	82217	Arg463Leu	–	–	–	Ala187Val	−79C>A^*b*^^,*f*^	Tyr137Asp^*b*^^,*f*^/−28T>C	Glu194fs	–	>16
High	83528	–	–	–	–	Ala187Thr^*b*^^,*f*^	–	–	–	Met31Ile	>16

^*a*^ Variants were classified according to the WHO mutation catalogue (2023) into five groups.

^*b*^ Group 3: uncertain significance.

^*c*^ Variant allele frequencies were categorized as ≤25%.

^*d*^ 25%–75%.

^*e*^ 75%–90%.

^*f*^ >90%.

^*g*^ Variants absent in the WHO mutation catalogue (2023) but documented in the TB-Profiler database.

^*h*^ Unlabeled mutations represent Group 4 (not associated with resistance–interim) or Group 5 (not associated with resistance) variants with allele frequencies >90%. MIC, minimum inhibitory concentration; fs, frameshift.

^*i*^ "–" indicates that the mutation was not detected.

## DISCUSSION

In this study, we investigated the genetic determinants causing the discrepancies between pDST and gDST for INH in clinical MTBC strains. Our results showed that 53 strains harbored 83 variants across nine genes associated with INH resistance. However, only five strains carried variants included in WHO catalogue Groups 1 and 2 (Table 1), specifically *katG* Ser315Asn, *katG* Trp39STOP, *inhA* −154G>A, and *inhA* Ser94Ala, which should be interpreted as markers of clinically relevant phenotypic INH resistance (15). It also should be noted that all of these variants, including *katG* Ser315Asn, were not covered by the MTBDR*plus* probes, which may explain the observed discrepancies between pDST and gDST for INH.

Among 44 strains lacking Group 1 and 2 variants but carrying variants belonging to WHO catalogue Group 3 (Table 2), two strains (32134 and 69442) also carried variant *katG* Val1Ala, which is not listed in the WHO catalogue but included in TB-Profiler. This variant is located at the N-terminus of KatG and may reduce KatG levels and peroxidase activity (16). Since INH is a prodrug that needs to be activated by KatG (17), we speculated that the low levels of KatG might lead to phenotypic INH resistance.

Previous studies have demonstrated that INH resistance is mainly associated with variants in *katG*, *inhA* and its promoter region, and *ahpC* and its promoter region (18). Among the 44 strains here, most carried variants in these genes or their promoter regions (Table 2). Notably, the *katG* gene and the promoter region of *ahpC* exhibited the highest variant frequencies; however, the association of these variants with phenotypic INH resistance remains unclear based on the current WHO mutation catalogue (15). Further investigation is required to elucidate the role of these variants, particularly those in the *katG* and the *ahpC* promoter region, in conferring INH resistance.

A nationwide survey conducted in China in 2007 on the prevalence of drug-resistant tuberculosis reported that mutations in the *ahpC-oxyR* intergenic region were identified in 18.6% (35/188) of INH-resistant strains (19). Notably, 85.7% (30/35) of these strains also carried additional mutations in *katG* or other genes. Nevertheless, the potential regional significance of ahpC-oxyR intergenic region mutations in the mechanisms of INH resistance remains an important consideration. However, widely used molecular assays like MTBDR*plus* are limited to detecting variants in the *katG* and *inhA* genes, excluding *ahpC*. This study highlights the potential value of detecting *ahpC* variants and suggests that MeltPro TB, which can detect *ahpC* variants (20), may be a more comprehensive and suitable test for accurately identifying INH resistance in China (20).

In addition to these well-characterized variants, other variants in genes, including but not limited to *efpA*, *fadE24*, *kasA*, *nat*, *dnaA*, *Rv0010c*, *mshA*, *hadA*, *Rv1129c*, *Rv1258c*, *ndh*, *Rv2752c*, and *glpK*, are also found to be associated with INH resistance (21–27). While the specific biological functions of some of these genes remain incompletely understood, they are hypothesized to play roles in influencing drug metabolism, altering drug targets, and modifying cell wall synthesis, which might affect INH drug resistance or tolerance. For example, variants in *kasA*, a gene involved in meromycolic acid biosynthesis, can reduce INH binding to its target, leading to resistance (28). Similarly, variants in *ndh*, which encodes NADH dehydrogenase, may inhibit INH activation by KatG, contributing to tolerance (29). In this study, variants in *mshA*, *dnaA*, *Rv0010c*, *Rv1129c*, *Rv1258c*, and *Rv2752c* were detected, suggesting their potential involvement in INH resistance pathways.

Our results showed that strains often carry multiple variants associated with INH resistance, and we identified 15 specific variants (e.g., *katG* Arg463Leu) that are present in both high- and low-level INH-resistant strains. This overlap makes it difficult to identify variants that specifically confer high or low levels of INH resistance (30). Previous studies have suggested that the accumulation of variants in genes, such as *katG*, *inhA*, and *ahpC*, may correlate with increased INH resistance (19, 31). However, our analysis comparing the number of variants between the high- and low-level INH-resistant strains using a *t*-test did not show a statistically significant difference (*t* = 0.231, *P* = 0.818). It should be noted that the resistance levels in this study were quantified using the MIC testing method, which was not intended to validate phenotypic DST classifications. For instance, six strains (20330, 25707, 25949, 28802, 30918, and 78704) with INH MICs of 0.125  µg/mL were found to be phenotypically resistant by MGIT 960 testing, yet were classified as susceptible based on MIC testing according to the WHO-recommended critical concentration (13). This discrepancy likely reflects inherent methodological differences between the two testing approaches.

The limited sensitivity of the current molecular methods for detecting heteroresistance, e.g., the MTBDR*plus* assay requires resistant subpopulations to exceed 5% for reliable identification (12), makes heteroresistance a critical factor contributing to discrepancies between pDST and gDST. In this study, WGS revealed 21 variants with frequencies ranging from 10.8% to 99.0% across 18 strains (34.0%, 18/53), indicating the prevalence of heteroresistance in this population. However, in the case of strain 72043, while sequencing revealed a *katG* Ser315Asn variant at 42% frequency, the MTBDR*plus* assay failed to detect INH resistance. This discrepancy likely reflects technical limitations of the assay rather than true heteroresistance (11). These findings highlight the need for more sensitive diagnostic tools to accurately detect heteroresistance in clinical strains (21, 32).

Our results also showed that six strains exhibited a genetic distance of fewer than 12 SNPs, suggesting recent transmission. However, epidemiological investigations and case interviews did not confirm direct transmission links among these individuals. Notably, four variants, including *katG* Arg463Leu, *Rv1129c* −28T>C, *Rv1258c* Glu194fs, and *mshA* Ala187Val, were present exclusively in lineage 2 strains. This lineage-specific distribution suggests distinct genetic adaptations and selective pressures between the lineage 2 and lineage 4 populations (33).

This study has several limitations. First, the single-center design and relatively small sample size may restrict the generalizability of our findings. Second, our analysis focused on genes listed in the WHO variant catalogue, potentially leading to the oversight of other resistance mechanisms, such as efflux pump overexpression (e.g., Rv1218c) (34, 35). Finally, although WGS identified multiple resistance-associated variants, the functional interactions among these variants remain unclear and necessitate further investigation through gene knockout and complementation studies.

In conclusion, our study revealed that the variants classified as “Group 3: uncertain significance” may be the main genetic determinants causing discordant INH DST results (INH-resistant by MGIT but INH-susceptible by MTBDR*plus*), highlighting their associations with INH resistance that need to be further investigated. In addition, this study emphasizes the importance of integrating advanced sequencing tools into DST to improve the accuracy of INH resistance detection, ultimately enhancing clinical decision-making and supporting the global fight against drug-resistant TB.

## Data Availability

The raw sequencing data have been deposited in the NCBI SRA under BioProject accession PRJNA1289107.

## SUPPLEMENTAL MATERIAL

Table S1 spectrum.01101-25-s0001.docx Mutation profiles and INH MIC values of 4 INH-resistant MTB strains lacking Group 1-3 variants.

ASM does not own the copyrights to Supplemental Material that may be linked to, or accessed through, an article. The authors have granted ASM a non-exclusive, world-wide license to publish the Supplemental Material files. Please contact the corresponding author directly for reuse.
