# Supplementary material for: Discrepancies in isoniazid susceptibility profiles: Bactec MGIT 960-resistant but GenoType MTBDRplus-susceptible Mycobacterium tuberculosis strains in Hunan, China
Source: Microbiol Spectr. 2025 Oct 1;13(11):e01101-25. doi: 10.1128/spectrum.01101-25 (PMC12584712; doi:10.1128/spectrum.01101-25)
Supplement: Table S1 — Mutation profiles and INH MIC values of 4 INH-resistant MTB strains lacking Group 1-3 variants. [file spectrum.01101-25-s0001.docx]

Table S1 Mutation profiles and INH MIC values of 4 INH-resistant MTB strains lacking Group 1-3 variants

| Resistant level | sample ID | *katG* | *mshA* | *Rv1129c* | *Rv1258c* | *Rv2752c* | MIC(µg/mL) |
| --- | --- | --- | --- | --- | --- | --- | --- |
| Low | 25725 | Val1Ala^△, g^/Arg463Leu | - | -28T>C | - | - | 0.5 |
| Low | 28427 | Arg463Leu | Ala187Val | -28T>C | Glu194fs | - | 0.5 |
| High | 21542 | Arg463Leu | Ala187Val | -28T>C | Glu194fs | - | 2 |
| High | 80067 | - | - | - | - | Met31Ile | 4 |

^△^Variants absent in WHO mutation catalogue (2023) but documented in TB-Profiler database.

Variant allele frequencies were categorized as: *^a^*>90%. Unlabelled mutations represent Group 4 (Not associated with resistance–interim) or Group 5 (Not associated with resistance) variants with allele frequencies >90%. MIC, minimum inhibitory concentration. fs, frameshift.
